# Molecular Ecological Insights into Neotropical Bird–Tick Interactions

**DOI:** 10.1371/journal.pone.0155989

**Published:** 2016-05-20

**Authors:** Matthew J. Miller, Helen J. Esser, Jose R. Loaiza, Edward Allen Herre, Celestino Aguilar, Diomedes Quintero, Eric Alvarez, Eldredge Bermingham

**Affiliations:** 1 Smithsonian Tropical Research Institute, Panama City, Republic of Panama; 2 Department of Biology, Villanova University, Villanova, Pennsylvania, United States of America; 3 Department of Environmental Sciences, Wageningen University, Wageningen, the Netherlands; 4 Centro de Biodiversidad y Descubrimiento de Drogas, Instituto de Investigaciones Científicas y Servicios de Alta Tecnología (INDICASAT-AIP), Panama City, Republic of Panama; 5 Centralamerican Master’s Program in Entomology, University of Panama, Panama City, Republic of Panama; 6 G. B. Fairchild Invertebrate Museum, University of Panama, Panama City, Republic of Panama; 7 Patricia and Phillip Frost Museum of Science, Miami, Florida, United States of America; Onderstepoort Veterinary Institute, SOUTH AFRICA

## Abstract

In the tropics, ticks parasitize many classes of vertebrate hosts. However, because many tropical tick species are only identifiable in the adult stage, and these adults usually parasitize mammals, most attention on the ecology of tick-host interactions has focused on mammalian hosts. In contrast, immature Neotropical ticks are often found on wild birds, yet difficulties in identifying immatures hinder studies of birds’ role in tropical tick ecology and tick-borne disease transmission. In Panama, we found immature ticks on 227 out of 3,498 individually–sampled birds representing 93 host species (24% of the bird species sampled, and 13% of the Panamanian land bird fauna). Tick parasitism rates did not vary with rainfall or temperature, but did vary significantly with several host ecological traits. Likewise, Neotropical–Nearctic migratory birds were significantly less likely to be infested than resident species. Using a molecular library developed from morphologically–identified adult ticks specifically for this study, we identified eleven tick species parasitizing birds, indicating that a substantial portion of the Panamanian avian species pool is parasitized by a diversity of tick species. Tick species that most commonly parasitized birds had the widest diversity of avian hosts, suggesting that immature tick species are opportunistic bird parasites. Although certain avian ecological traits are positively associated with parasitism, we found no evidence that individual tick species show specificity to particular avian host ecological traits. Finally, our data suggest that the four principal vectors of Rocky Mountain Spotted Fever in the Neotropics rarely, if ever, parasitize Panamanian birds. However, other tick species that harbor newly–discovered rickettsial parasites of unknown pathogenicity are frequently found on these birds. Given our discovery of broad interaction between Panamanian tick and avian biodiversity, future work on tick ecology and the dynamics of emerging tropical tick-borne pathogens should explicitly consider wild bird as hosts.

## Introduction

Wild birds are increasingly recognized as playing an important role in human and animal health. Emerging zoonotic diseases such as avian influenza, West Nile Virus, and Lyme disease often have wild birds in their transmission cycle [1–3], and wild birds can act as reservoir hosts in endemic areas [4]. Birds also have the potential to introduce diseases into previously naïve populations by spreading pathogens and/or their vectors over large distances via migratory flyways [5,6]. Hard ticks (Ixodidae) are haematophagous ectoparasites regularly found on wild birds, providing them habitat and blood-meal resources, and vector more human pathogens than any other arthropod group [7]. Migratory birds captured in temperate regions upon their return from wintering grounds have been observed infected with larval and nymphal stages of tropical tick species [5]. The ability of migratory birds to move tropical ticks over long distances, in concert with global climate change [8], potentially exposes extra-tropical regions to novel tropical tick-borne pathogens and vice versa [7,9,10]. Therefore, empirical studies are needed that evaluate which tick species are frequently involved in bird parasitism and which ecological characteristics of bird species are related to increased tick infestation levels.

In the New World tropics, the greatest risk of tick-borne disease for human comes from *Rickettsia rickettsii*, the etiological agent of Rocky Mountain Spotted Fever (RMSF), which has a current fatality rate of 20–40% [11]. In the Neotropics, the principle vector of RMSF are members of the *Amblyomma cajennense sensu lato* species complex [12], although other species of ticks have been show to harbor *R*. *rickettsii*. In Panama, four confirmed RMSF vectors occur: *A*. *mixtum*, which is the local taxon in the *A*. *cajennense* species-complex [13], along with three other species which are widely found in the Neotropical region: *Dermecantor nitens*, *Haemaphysalis leporispalustris*, and *Rhipicephalus sanguineus* [14]. Although the first clinical cases of RMSF were reported in Panama in the 1950s, the disease went unreported for over 50 years until a fatal case in 2004 [15]. In the past decade, additional fatal cases have been reported in Panama [16] as well as adjacent Costa Rica [17] and Colombia [18]. Improvements in diagnosis are clearly responsible for at least part of the surge in recent cases, although climate change, habitat modification, and/or increases in human–wildlife contact may also be responsible [19]. While adults of the genus *Amblyomma* and several other Neotropical tick species typically exploit mammals, or reptiles and amphibians to a lesser degree [20], immature forms are routinely found on birds [7,20–26] including, rarely, nymphs of the *A*. *cajennense* species complex [27].

At the same time, improvements in molecular detection of tick endosymbionts has uncovered a diversity of novel *Rickettsia* strains of unknown pathogenicity in Neotropical ticks [21,28,29], and wider distributions of other *Rickettsia* species known to cause RMSF-like symptoms. Recently, *R*. *rickettsii* has been found in a variety of tick species throughout the Americas and is believed to have caused many overlooked cases of rickettsial disease in South America [19]. Rickettsial bacteria have been found in several species of ticks recovered from Neotropical birds [21,25,26].

Our understanding of Neotropical bird-tick associations and hence their role in disease transmission has been hampered by species identification problems; most immature Neotropical ticks, especially those of the genus *Amblyomma*, are not readily identifiable to species by morphology alone. *Amblyomma* species diversity peaks in the Neotropics, where taxonomic keys serve to identify the nymphal stage of few *Amblyomma* species [30]. For example, in a recent survey of ticks found parasitizing humans in Panama, only 38% of the recovered specimens of *Amblyomma* could be identified to species [31]. Similarly, in a study documenting tick infestation patterns in wild birds from southeastern Brazil, nearly 48% of the specimens of *Amblyomma* could not be identified to species [27]. That study and others [21,32] demonstrate that new tools and approaches are essential to properly assess the role of wild birds in tick ecology and tick-borne disease transmission in the Neotropics.

Museum specimens are often valuable resources for broad studies of ecological patterns [33]. Here, we exploit seven years of extensive bird specimen collection across Panama to clarify the ecological interactions between the diverse tick and bird fauna of this Neotropical country. The collecting program of the STRI Bird Collection visited over 100 field sites, sampling nearly 3500 terrestrial birds from 384 species, almost half of the roughly 800 non-aquatic bird species recorded from Panama. Our study provides an unparalleled insight into the ecological relationships between birds and ticks in a Neotropical setting. Specifically, our goals were to: 1) identify how avian ecological traits influence the frequency of tick parasitism of Panamanian bird species; 2) produce a robust DNA barcode library capable of identifying most of the commonly encountered immature ticks in Panama; and 3) use the DNA barcode library to identify to species immature ticks collected off of Panamanian wild birds to determine host specificity patterns and the role of parasite ecological filtering in shaping bird-tick associations in Panama. Our results should provide insights into the relation between avian and ixodid biodiversity that may better inform our understanding of Neotropical tick ecology and may provide insights for our understanding of emerging tropical tick-borne diseases.

## Results

### Ecological traits of Panamanian wild birds parasitized by ticks

We evaluated patterns of tick parasitism in 3,498 bird specimens from the STRI bird collection, representing 384 species, i.e. nearly half of the roughly 800 non-aquatic bird species recorded from Panama. Ticks parasitized a total of 227 specimens of Panamanian birds (6.5% of all individuals; S1 Table) representing 93 avian species and 24 families. Among the 227 infested birds, both the median and modal number of ticks recovered was 1. However, 15% of the infested birds had 4 or more ticks; one bird had 101 ticks, which was the most recovered from any bird in the study. Resident birds (i.e. species that breed in Panama) were 3.8 times as likely to be parasitized by ticks compared to non-breeding Nearctic–Neotropical migratory species (6.8% vs. 1.8%; Fisher’s exact test, *P* = 0.001). 90 of 343 resident species had at least one bird sampled infested with ticks, whereas only 3 of 40 Nearctic–Neotropical migratory species had an infested individual, although sample sizes were lower for migratory species relative to resident species (mean *N*_*resident*_ = 9.5, mean *N*_*migratory*_ = 5.4; two-tailed unequal variance t-test, *P* < 0.001).

Among avian families with at least 25 specimens evaluated, the families with the greatest proportion (%) of specimens parasitized by ticks were: Thamnophilidae (antbirds: 18%; 12 of 20 species); Furnariidae (ovenbirds and woodcreepers: 15%; 11 of 20 species); Polioptilidae (gnatcatchers: 15%; 1 of 3 species); Turdidae (thrushes: 15%; 7 of 15 species); and Troglodytidae (wrens: 14%; 9 of 16 species). Because of behavioral differences between males and females (including time spent in the nest, where infestation may be more likely), we anticipated a difference in infestation rates between males and females. Although females had a slightly higher prevalence of tick parasitism than males (7% vs. 6%), the difference was not significant (Fisher’s exact test, *P* = 0.23). Among resident birds, a logistic model indicated that only forest habitation, terrestrial foraging, bark insectivory, and lowland residency were significantly positively associated with tick parasitism (Table 1).

**Table 1:** Ecological traits associated with tick parasitism among Panamanian resident wild birds (*N* = 3274).

Ecological Trait	Odds Ratio	*P* value (Wald’s)
Bark insectivores*	8.3	0.004
Terrestrial foragers*	4.0	< 10^−15^
Forest vs. non-forest dwellers*	3.3	< 10^−9^
Tree hole nesters	2.5	0.20
Ground cavity nesters	2.0	0.14
Lowland vs. montane*	1.7	0.01

Traits significant for a positive association with tick parasitism marked with (*). Significance determined by multiple logistic regression.

Among 26 locations where we sampled a minimum of 20 birds, we found no relationship between the frequency of tick parasitism and annual mean temperature, temperature seasonality, annual precipitation, or precipitation seasonality. However we did recover an effect of taxonomic composition, specifically, the proportion of the sampled avifauna belonging to the five families most frequently parasitized by ticks (S1 Table).

### DNA identifications of adult ticks agree with morphological taxonomy

The 96 individuals in the adult reference library of morphologically identified ticks from central Panama formed 20 clusters with pairwise Kimura-2 parameter (K2P) genetic distances greater than 5% (S1 Fig). All 96 could be placed in clusters in agreement with the original morphological identification of the voucher with bootstrap support values of at least 99%. Among the 20 species—clusters, average nearest-neighbor K2P distance to another cluster was 15.6% and the minimum nearest-neighbor K2P distance was 12.5% (range: 12.5%– 20.5%).

Two species contained DNA barcode sub-clusters with between-cluster sequence divergence well below the 12.5–20.5% difference observed between named species, but greater than 3.0%. *Haemaphysalis juxtakochi* comprised two clusters that differed by 3.0% pairwise K2P distance, with each cluster supported by 99% bootstrap support, while *Amblyomma ovale* contained one cluster of four individuals supported by 97% bootstrap and a fifth individual that varied by an average K2P distance of 3.2%. In both cases, individuals from both clusters were collected at a shared location, suggesting that these might represent cryptic biological species. As a consequence, a numerical DNA barcoding taxonomy (BIN barcode identification number) based on genetic distances among barcodes clusters recovered 22 unique BINs in our adult dataset; representing the 20 clusters that agree with our morphological named species as described above, as well as second BINs for both *H*. *juxtakochi* and *A*. *ovale*.

### DNA identifications of immature ticks from birds

We generated useable DNA barcode sequences from 130 immature ticks out of a total of 172 samples attempted (76% success rate). Two failures were due to double peaks in the electropheragram recovered in multiple amplification and sequencing attempts (MJM2941-T01 and MJM4264-T01); we removed these individuals from further analyses. One individual (MJM 7015) amplified its avian host (*Poecilotriccus sylvia*) DNA. The remaining 39 individuals failed in either the PCR or the sequencing step (S1 Table).

**Fig 1:**
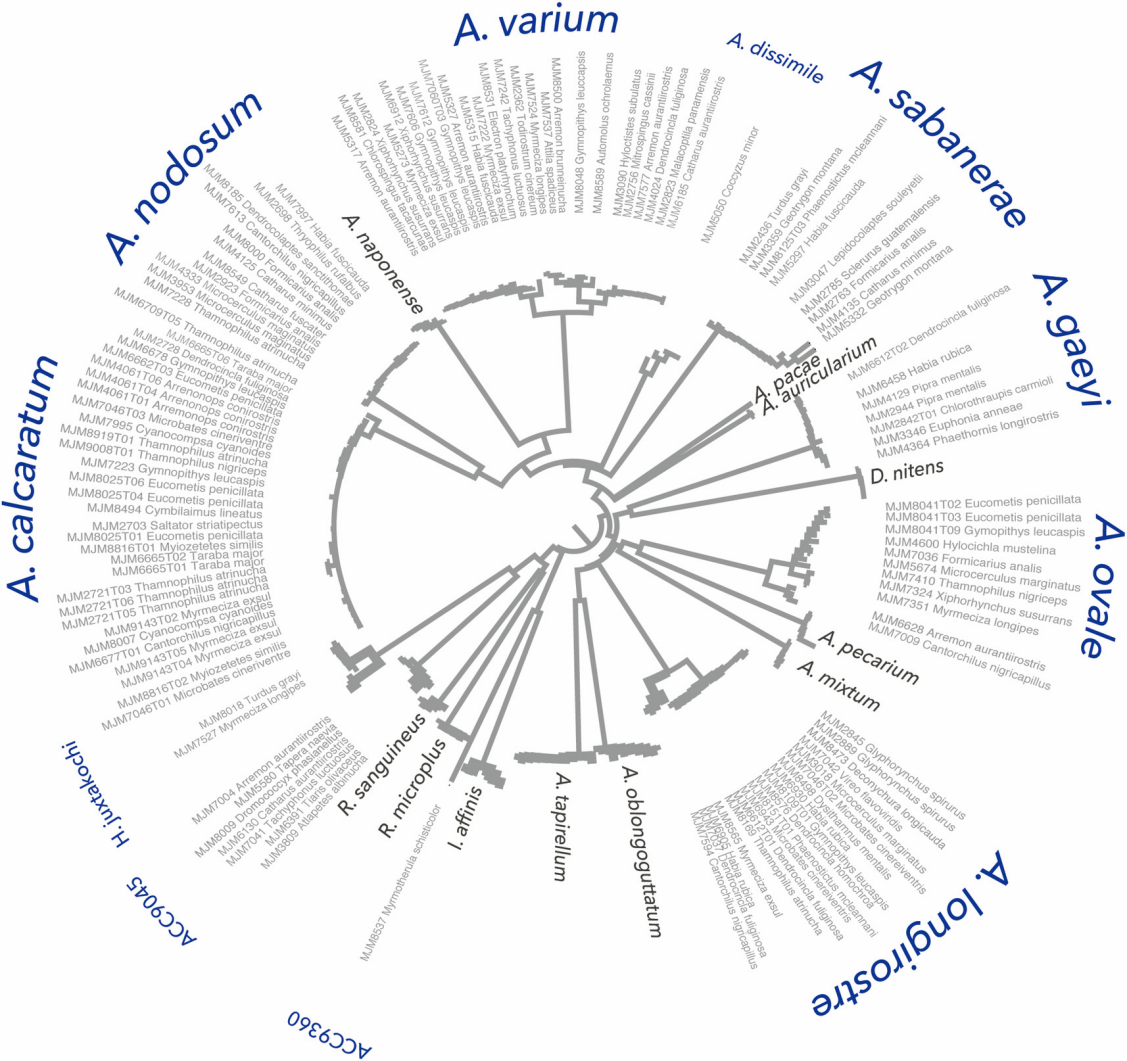
DNA barcoding neighbor-joining tree of combined data matrix of immature ticks and adult reference library ticks. Thin gray tip labels refer to specimen number and host species. Clade labels in blue refer to tick species recovered from birds, clade labels in gray refer to ticks unobserved on birds. Two clades where not represented by in the adult reference library so they are labeled in red with their molecular taxonomy Barcode Identification Number (BIN). The distribution of Panamanian ticks recovered from wild birds is unbalanced towards certain species of *Amblyomma*. Importantly, we recovered no immature ticks on birds from species known to vector human disease [6].

Sequences from the immature ticks formed 13 DNA barcode clusters. When we merged the immature barcode dataset with the adult reference library (Fig 1), 122 of 130 (94%) taxa formed 11 DNA barcode clusters which included an adult reference. Thus, we can confirm that immature ticks of the following species parasitize wild birds in Panama: *H*. *juxtakochi*, *A*. *dissimile*, *A*. *ovale*, *A*. *longirostre*, *A*. *geayi*, *A*. *sabanerae*, *A*. *varium*, *A*. *calcaratum*, and *A*. *nodosum* (S1 Table). Our data establish that at least 8 of the 18 species of *Amblyomma* ticks found in Panama parasitize birds. This includes the second global record of *A*. *dissimile–*a reptile and anuran specialist–parasitizing a wild bird (Mangrove Cuckoo, *Coccyzus minor*, [20]). In the case of *H*. *juxtakochi*, which is represented in the adult reference library by two DNA barcode clusters (and two unique BINs in the molecular taxonomy), we recovered both clusters from bird samples. The two remaining clusters of immature ticks did not include an adult reference, so they can only be identified using molecular taxonomy (see below).

Using the BIN molecular taxonomy, our sample of immature ticks clustered into 13 BINs, including 11 of the 22 BINs recovered in the adult reference library. Hence, we were able to assign a BIN to 100% of the samples that provided DNA sequences and to 76% (130 of 172) of all samples for which we attempted DNA barcoding. Eight other immature ticks (6%) formed two novel clusters on our phylogenetic trees; and for these we were only able to assign a BIN numerical taxonomic identification. The first BIN (ACC9360) was formed by just one immature tick that had a nearest neighbor-distance of 14.4% to the reference library cluster of *Ixodes affinis*. A second cluster (BIN: ACC9045) was formed by seven immatures whose nearest-neighbor cluster on the BOLD database did not include ticks from this study. Instead, they were most closely related to *H*. *leporispalustris* collected in Canada, with a nearest-neighbor distance of 6.0%. *H*. *leporispalustris* also occurs in Panama, but without a Panamanian adult reference sequence and given the large sequence variation, we are unable to determine whether our sample represents a genetically-divergent Panamanian *H*. *leporispalustris* population or distinct species of *Haemaphysalis* yet to be recorded in Panama. Thus, we refer to these two taxa as probable members of *Ixodes* and *Haemaphysalis* genera, respectively.

**Fig 2:**
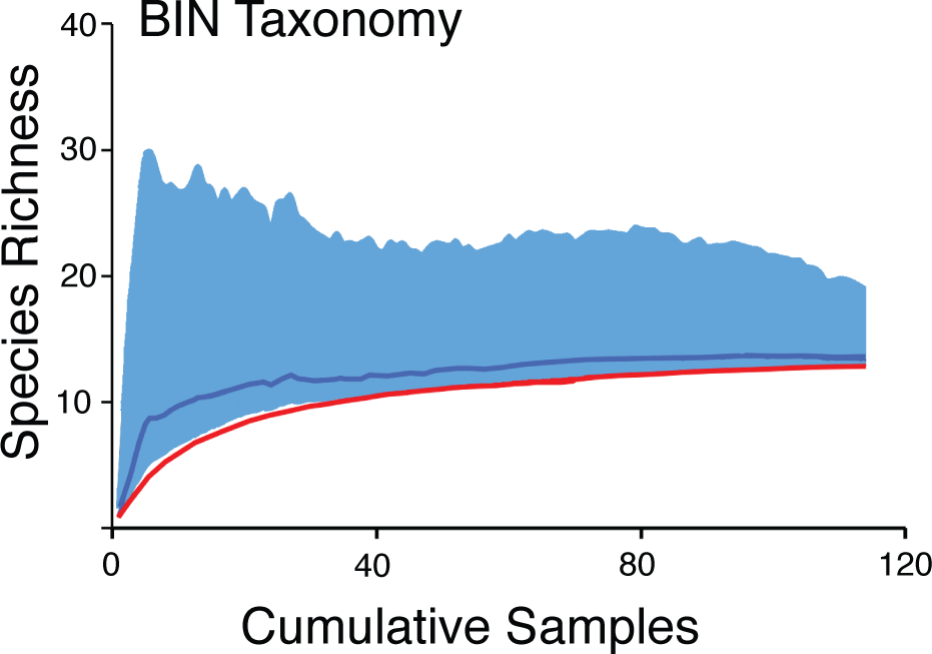
Species accumulation curve (SAC) for immature ticks recovered from Panamanian wild birds based on BIN numerical taxonomy (see S2 Fig for SAC based on traditional tick taxonomy). Red line = *S*, mean observed species richness; dark blue line = *Ŝ*, mean Chao1 *S* estimate; filled blue area defines 95% upper and lower confidence limits (CI) for *Ŝ*. As Chao1 is downward biased, the 95% lower CI is probably not useful. The convergence of the S and *Ŝ* curves, as well as the asymptotic nature of these curves and the CI curves, suggests that at most only a few more tick species would be recovered from wild birds in Panama, and that their occurrence would be rare.

Using either BIN or traditional taxonomy, species accumulation curves for our sample of immature ticks recovered from wild birds were essentially asymptotic, as were species richness estimators designed to account for unobserved species. Using the BIN taxonomy, the Chao1 species richness estimate was 13.3 (95% confidence interval: 13.0–19.0), compared to an observed BIN species richness of 13 (Fig 2). Likewise, the species accumulation curve using the traditional taxonomy recovered a mean Chao1 estimate of 11.5 (95% confidence interval: 11.0–19.3), compared to an observed species richness of 11 (S2 Fig). These results suggest that at most only a few more tick species would be recovered from wild birds in Panama given considerably greater sampling effort, and that their occurrence on wild birds would be exceedingly rare.

### Immature ticks show no species-level host specificity or ecological filtering of avian hosts

Immature tick species showed no measurable avian species host specificity. Tick species collected from at least two different bird individuals always occurred on at least two different avian host species, and most frequently occurring tick species recovered from wild birds in our samples had the greatest number of avian host species (Pearson’s *rho* = 0.984, *P* < 0.0000001). In 5 of 11 birds from which we sequenced multiple individual ticks, we found more than one species or more than one haplotype of tick, suggesting multiple independent colonizations of the host.

**Fig 3:**
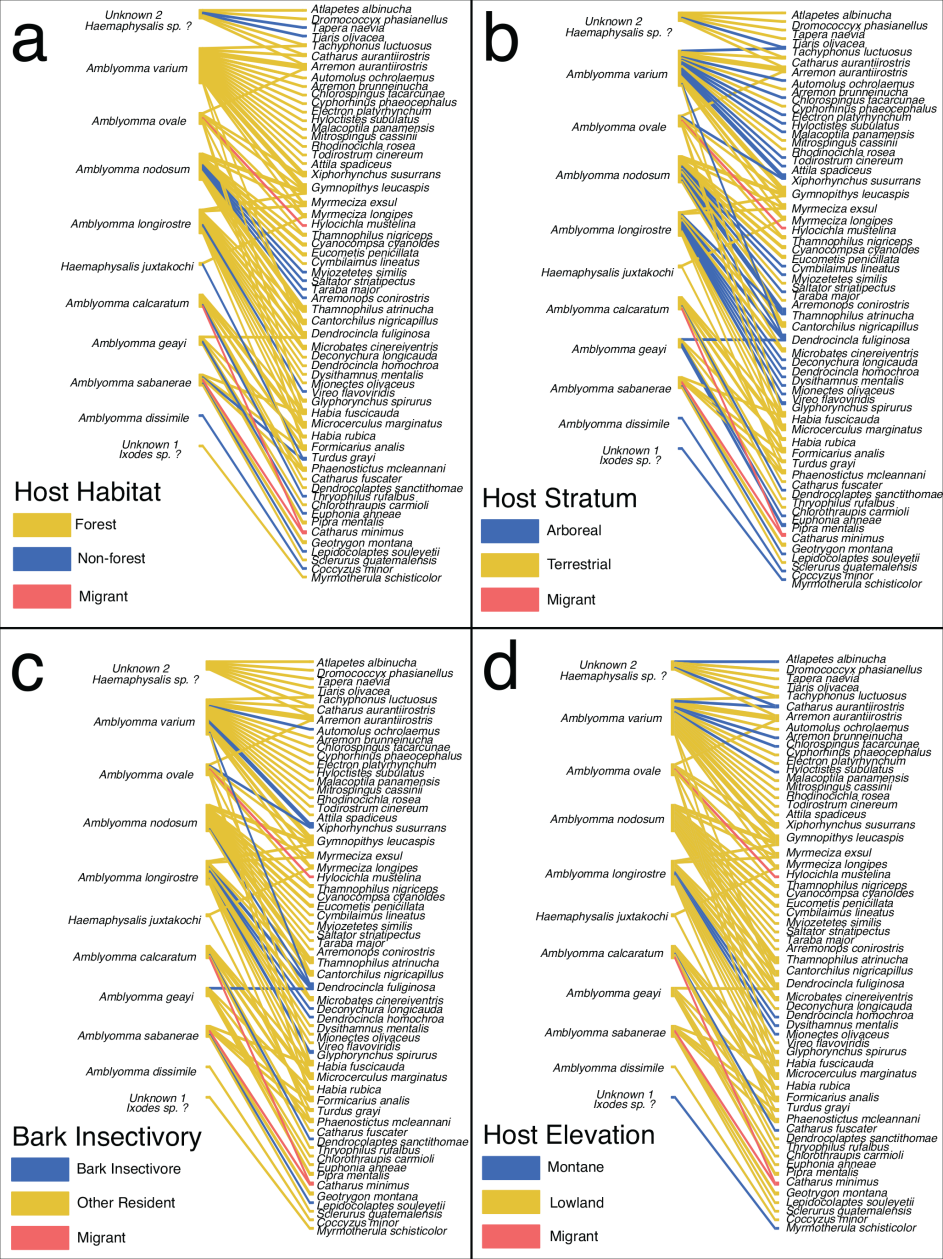
Bird–immature tick quantitative interaction networks. (A) Blue: interactions involving non-forest bird species, yellow: interactions involving forest inhabiting bird species; (B) yellow: terrestrial-foraging bird species; blue: interactions involving arboreal bird species; (C) blue: interactions involving bird species that are bark insectivores; yellow: other species; (D) blue: interactions involving montane bird species, yellow: lowland species. a-d) pink: interactions involving non-breeding migrant bird species. The frequency of hosts among these four ecological traits was not significant among the 11 sampled tick species (See Results for details).

In addition to the lack of species-level host specificity, we observed little evidence that individual tick species show ecological preferences in hosts. Instead, networks of specific tick and avian host species demonstrates broad and varied interactions by the ecological traits that were previously shown to influence rates of bird parasitism (Fig 3). We found no difference in the frequency of forest versus non-forest hosts among our 13 tick BIN species (*G* = 12.37, d.f. = 12, *P* = 0.42, Fig 3A), nor in the frequency of arboreal-foraging hosts (*G* = 22.18, d.f. = 12, *P* = 0.036, Bonferonni-corrected α = 0.013, Fig 3B). Likewise, neither the proportion of bark insectivorous hosts (*G* = 10.287, d.f. = 12, *P* = 0.59, Fig 3C) nor the proportion of montane hosts (*G* = 17.996, d.f. = 12, *P* = 0.12, Fig 3D) varied among tick species. We found equivalent results using the traditional tick taxonomy.

## Discussion

### Patterns of tick parasitism on Panamanian wild birds

Although only a minority of Panamanian land birds are infested by ticks, the broad participation in bird–tick interactions by a diversity of bird and tick taxa indicates that wild birds may play an important role in the life history of many Neotropical tick species and have the potential to play a role in the transmission of tick-borne diseases. While only 6.5% of all birds examined carried ticks, parasitism by ticks includes a diverse taxonomic array of avian hosts. We observed ticks on 93 of the 384 species we examined, which represent 24 avian families. When examining parasitism rates for passerines (11.2%), the Panamanian parasitism rate is in line with rates (9–17%) from other studies that focused almost exclusively on passerine birds [22,32,34].

We found no evidence that tick parasitism by site varied by either absolute or seasonal differences in temperature or rainfall. This indicates that, at the macro-scale, climatological patterns in the Neotropics likely have little influence on the parasitism frequency. Nonetheless, differences in ecological traits among Panamanian birds appear to modulate tick parasitism frequency. We found that lowland, forest inhabiting, ground and bark foraging birds were significantly more likely to be infested with ticks than birds with other ecologies.

### Lack of host specificity and ecological filtering in bird-tick interactions

Historically, it has been argued that adult life stages of Neotropical ticks are strictly or nearly-strictly specific to one or a few mammalian species [20,35,36]. The degree of host specificity in immature Neotropical ticks remains largely unexplored, because of the difficulty of their morphological identification to species. Using our molecular identifications, we found no evidence for strong host specificity in Panamanian tick–bird interactions. Instead, the number of host species increased with the number of sampling events for that tick species, demonstrating that immature Neotropical ticks parasitize a wide variety of birds from diverse families, with no clear preference for particular host species.

Our finding of limited or no host specificity tracks a more general shift in our understanding of the specificity of tick–host relationships. Researchers applying modern statistical approaches generally have found lower levels of host specificity than was assumed in the historical literature [37–40]. Instead of absolute host specificity, research suggests that ecological correlates between potential hosts and ticks may determine the host diversity of tick species [37]. This is a restatement of Combe’s “ecological filter” concept of parasitism [41,42]. Our finding of four key ecological traits that are positively correlated with tick parasitism rates in birds allows us to test the ecological filter concept in bird–immature tick interactions.

Here, we found no evidence that certain tick species have ecological preferences to bird species based on any of these four traits. The result was robust using both traditional tick taxonomy and the molecular taxonomy. This latter result is notable because one of the key ways that cryptic tick species might co-occur is by having cryptic host ecological specificities among immature stages [43]. Whether such ecological filtering occurs between adult ticks and mammals in Panama awaits further study. Our finding of neither strict-host specificity, nor the more relaxed ecological filtering type of specificity in bird–tick interactions, coupled with the diversity of bird species involved and the potentially greater dispersal ability of birds, indicates that bird–tick interactions will have meaningful implications for the demographics of Neotropical ticks and also the ecology of tick-borne pathogens [37]. Thus, tick-borne disease transmission models based on patterns of host specificity of adult ticks may require re-examination in order to incorporate the more labile ecology of immature ticks.

### DNA barcoding of adult and immature ticks

Our findings demonstrate that DNA barcodes are a reliable method to identify Panamanian hard ticks (Ixodidae) to species, and can overcome the frequently-cited difficulties in identifying immature forms of Neotropical ticks to species using only morphological characters [22,23,44]. Alternatives to molecular identification of immature ticks include rearing immatures to life stages that can be reliably identified to species [27], but this is time consuming, requires special laboratory conditions that vary among species, and most immature ticks die before reaching an identifiable life stage [23,27]. DNA barcoding appears to yield a much greater percentage of successful species identifications. We were able to identify ~70% of immature ticks, whereas a rearing study from Brazil was able to identify only 12% of the immatures [23].

### The role of wild birds in the transmission ecology of tick-borne pathogens

Our ability to identify immature ticks from Panamanian birds permits insight to the potential role of wild birds in the transmission ecology of Neotropical ticks and tick-borne diseases. We found no evidence that wild birds are involved in the transmission ecology of Rocky Mountain Spotted Fever (RMSF). RMSF is the most virulent tick-borne disease known in the Western Hemisphere and is caused by infection from *Rickettsia rickettsii*. In Panama, RMSF was first reported over 60 years ago [45], although it remained unreported again until 2004 [15], when it resulted in a fatal case in western Panama. Since 2004, RMSF has been regularly reported in central and western Panama [46]. Several species of ixodid ticks are confirmed vectors of *R*. *rickettsii*, including four species of Panamanian ticks: *Dermacentor nitens*, *Rhipicephalus sanguineus*, *Haemaphysalis leporispalustris*, and *Amblyomma *(*cajennense*) *mixtum*. However, members of the *A*. *cajennense* species complex are considered the primary vector of *R*. *rickettsii* in tropical America [12], and in Panama *R*. *rickettsii* has been detected principally in *A*. *mixtum* [47,48] with a single record in *D*. *nitens* [49]. We found no examples of *A*. *mixtum*, *D*. *nitens*, or *R*. *sanguineus* parasitizing birds in our study, although we did find five birds infested with *Haemaphysalis sp*. ticks. Our species accumulation curves (Fig 2, S2 Fig) for tick species found on wild birds suggest that at best only a few, rare, species are yet to be recovered from resident wild land birds in Panama. While a couple of studies from other Neotropical regions have reported that immature forms of species in the *A*. *cajennense* complex parasitize various wild bird species, Labruna et al. [27] challenged the morphological identifications in these cases. Our findings along with others [22,23,25,34] demonstrate that Rocky Mountain Spotted Fever vectors are at best rare parasites of wild birds, and collectively suggest a relatively negligible role for wild birds in Rocky Mountain Spotted Fever transmission in Panama and elsewhere in the Neotropics.

On the other hand, it is likely that immature ticks on wild bird in Panama harbor a diversity of other rickettsial pathogens. Although we did not attempt to isolate *Rickettsia* from sampled ticks, other studies have demonstrated that 10 of the 11 tick species that we recovered from Panamanian wild bird harbor *Rickettsia*. *Rickettsia amblyommii *has been recovered as a parasite of *A*. *ovale* in Panama [49,50] as well as *A*. *geayi* and *A*. *longirostre* in Brazil [22,29] and several species of ticks recovered from Neotropical–Nearctic migrant birds in south Texas, USA [51]. *Rickettsia amblyommii* has been suggested, but not confirmed, to be a cause of spotted fever-like disease in North America [52]. *Rickettsia parkeri*, which was recently identified as the cause of human spotted fever rickettsiosis in southeastern USA as well as in Brazil, Uruguay and Argentina was recovered from Brazilian samples of *A*. *nodosum* and *A*. *ovale* [29]. Other *Rickettsia *species, not yet known to cause human disease, have been recovered from *A*. *calcaratum* [21], *A*. *dissimile* [53], *A*. *varium* [54] and *H*. *juxtakochi* [29]. Work in the Neotropical regions concerning the pathogenicity of *R*. *amblyommii* and *R*. *parkeri* and other rickettsiales is in its infancy. Likewise, spotted fever group rickettsioses are often under-detected, especially in Middle and South America [54]. While the rate of infestation of ticks on wild birds in Panama is relatively modest (~6.5%), as a recent study from south Texas demonstrates, even low relative frequencies of parasitism may have regionally–important consequences for tick and emerging disease ecology given the absolute numbers of wild birds involved [51]. Assuming typical avian densities recorded for Panama [55], as many as 96,000,000 Panamanian wild birds might be infested with ticks. Thus, our finding of a pervasive and diverse relationship between birds and immature ticks in Panama suggests that further consideration of the role of wild birds in the ecology of Neotropical ticks and the pathogens they vector is warranted.

## Materials and Methods

### Bird and tick specimen collection

All bird records come from the vouchered collecting program of the STRI Bird Collection, conducted between 2008 and 2012. Collecting occurred year round; however as our collection strategy during this period was the developed as part of a larger program on the ecology of avian-mediated zoonoses, collecting was balanced at most sites between the rainy (May–December) and dry (January–April) season, as well as the across the migratory seasons of Nearctic–Neotropical migrants. Full specimen metadata are available in S1 Table and in the STRI Collections portal (http://stricollections.org).

**Fig 4:**
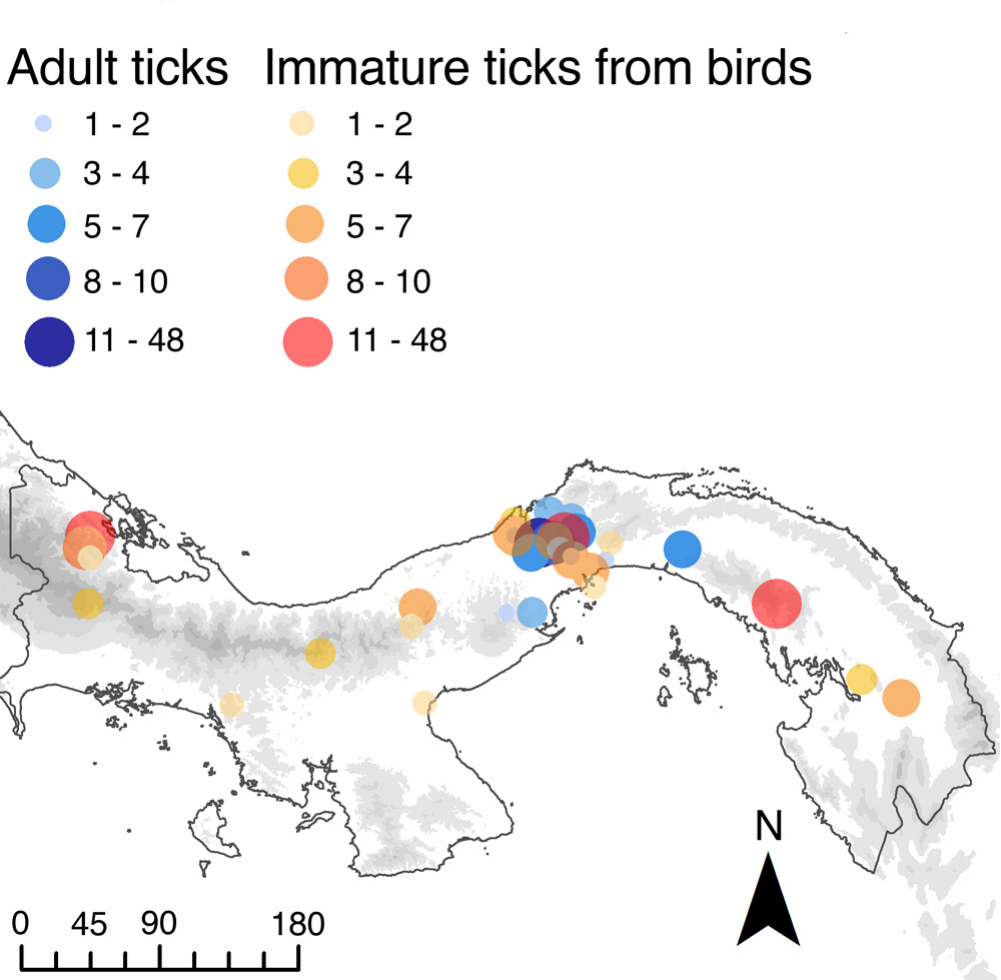
Map of collecting areas for adult and immature ticks. Blue circles represent areas where adult ticks were sampled for the reference library; orange circles represent locations where immature ticks were sampled from birds.

Our field procedures begin with capturing birds in mistnets (or occasionally collecting with shotgun). Per STRIBC field protocols, wild birds were euthanized in the field and flash frozen on solid CO_2_ in individual freezer bags to eliminate the risk of cross-contamination of ectoparasites prior to transportation to the lab. There, the entire ectoparasite assembly is recovered as the first step in the ornithological specimen preparation process via whole body ruffling following [56] who demonstrated that post-mortem ruffling is superior for estimating ectoparasite abundances compared to visual inspection and other live- bird sampling strategies. The number of sampling locations was more than 100, however we combined locations that were within 5 kilometers of each other, resulting in a total of 43 sampling locations throughout Panama (Fig 4) for the purpose of this study (S2 Table). EA separated ticks from other ectoparasites and identified all to age class and sex when possible. Ticks are stored in the STRI Cryological collections in single tubes per avian specimen in 95% ethanol and maintained at -20°C. All specimens collected by the STRI Bird Collection was done with the prior approval of ANAM, Panama’s environmental authority (permit numbers: SE/A-60- 10, SE/A-137-10, SE/A-96-09, SE/A-44-10, SE/A-66-11, SE/ A-2-12), and collecting methods have been approved by the Smithsonian Tropical Research Institute’s Institutional Animal Care and Use Committee (IACUC permits: 2007–03-03-15-07, 2011-0927-2014-03). Complete ornithological specimen metadata is available at: http://stricollections.org.

### Ecological patterns of tick parasitism on birds

We generated a tick parasitism data matrix (presence or absence of hard ticks: Ixodidae). Non-land birds from marsh, aquatic, and riverine habitats (e.g. Anseriformes, Charadriiformes, Alcedinidae) were excluded from the analysis, as well as aerial foragers (e.g. Apodidae, Hirundinidae), resulting in 3498 specimen records (S1 Table), from 384 avian species. Using this matrix, we analyzed whether key traits of the host species were correlated with tick parasitism using contingency table analysis. We considered the relationship of eight traits on tick parasitism, some of which have previously been correlated with tick parasitism in birds: residents vs. non-breeding migrants, females vs. males, terrestrial foraging vs. arboreal, ground cavity nesting, tree hole nesting, lowland vs. montane habitats, bark insectivory, forest vs. non-forest habitats. We evaluated the influence of sex and migratory status on the entire dataset, however because ecological traits for many migratory birds are more labile away from their breeding grounds and classifications based on temperate zone ecology may not reflect behavior in Panama, we evaluated the relationship between the remaining six traits and tick parasitism only on resident birds (i.e. those species that breed in Panama). Montane species were defined as those found almost exclusively 600 meters above sea level. Terrestrial foraging species include those species that forage primarily, but not exclusively, on the ground or within 15 centimeters of the ground. Forest inhabitants live in or require mature tropical forests; most edge species were classified as non-forest inhabitants. We evaluated all possible combinations of ecological characters using logistic regression models.

### Adult DNA Barcode Reference Library

We generated a DNA barcode reference library for the ticks of central Panama using morphologically pre-identified adult ticks collected as part of on-going research programs (HJE and JRL) on the tick-host interactions in the area surrounding the Panama Canal, and also in a few cases from ethanol preserved museum specimens of adult ticks (S1 Fig). Field collections were accomplished either by removal of ticks from hosts (live-captured animals, road kill, livestock, and pets) or by collecting questing ticks from the free environment via flagging, e.g. sweeping a white cotton cloth along vegetation and leaf litter and harvesting accumulated ticks. Adults were identified using morphological characters and existing taxonomic keys [20,57], and were stored in 95% ethanol and frozen at -20°C prior to molecular analysis (see next section). Our adult reference library included 96 individuals (S3 Table) that were morphologically assigned to 19 of the approximately 37 species of hard ticks recognized for the Republic of Panama, including 14 of 18 species of *Amblyomma* [20]. Unless already part of a museum collection, after DNA extraction adult reference ticks were stored in 95% ethanol and are maintained as voucher specimens in the ectoparasite collection of the STRIBC. A public dataset for these 96 specimens, including geographic details of collection, specimen photographs, and museum voucher information can be found on the BOLD data portal v3 [58] under the name: DS-TICKA (dx.doi.org/10.5883/DS-TICKA).

### Immature Ticks from Panamanian Wild Birds

We selected 186 immature ticks from the pool of immature ticks collected from birds for molecular species-level identification using the adult reference DNA barcode library as the basis for identification (S1 Table). We obtained usable DNA barcode sequences for 130 (see Results). Sample details for immature ticks can be found on the BOLD data portal under the name: DS-TICKI (dx.doi.org/10.5883/DS-TICKI).

### Molecular methods

To allow for the preservation of museum vouchers, DNA was extracted from adult specimens from either two legs removed from the specimen, or from a rear quarter section of the abdomen cut from the body, done under an entomological dissecting microscope. We used the entire body of immature ticks for DNA extraction. In all cases, the material being extracted was frozen in a 2 ml tube suspended in liquid nitrogen and pulverized using a sterile micro-pestle to improve DNA yield. We initially obtained poor DNA yield after attempted DNA extractions using DNAeasy spin columns (Qiagen, Valencia, CA), following the manufacturer’s instructions (except that we reduced the final elution volume to 50 μl). Subsequently, we switched to the QIAamp DNA Micro kit (Qiagen), which uses similar spin column technology but is optimized for smaller samples and resulted in superior DNA yields.

Amplification of the DNA barcoding region (5’ region of the COI mitochondrial gene, 43) was accomplished using the standard invertebrate primers (LCO1490 and HCO2198; 44) following [59], except that we halved the reaction volume (i.e. 25 μL) and raised DNA to 4 μL; we used Qiagen taq and buffers. Positive and negative controls were run in every reaction. Amplifications were visualized on a low-melting agarose gel from which a single PCR product was extracted using a sterilized scalpel blade, and sequenced at the Naos Molecular Laboratory, Smithsonian Tropical Research Institute. DNA sequences and tracefiles can be examined in BOLD under the DS-TICKA database and DS-TICKI database. Sequences have also been deposited in GenBank under accession numbers KF200076 –KF200171.

### Tree building and barcode distance analysis

In order to understand species limits and confirm morphological identification among our adult reference ticks (*N* = 96), we generated a neighbor-joining tree in MEGA v.5.1 [60] using Kimura-2 parameter (K2P) distances. We assessed branch support by bootstrapping the topology with 500 replicates. We examined the K2P distance matrix and resulting topology for evidence of genetic divergence among our adult reference library that might provide evidence for the presence of cryptic species [43] using both a standard genetic distance approach (3% K2P) as well as looking for the assignation of multiple Barcode Index Numbers (BINs) to a given species. The Barcode Index Number is an alternative, numerical taxonomy that clusters taxa into interim operational taxonomic units using a stage process to employ single linkage clustering [61]. BINs are assigned automatically in the BOLD database portal based on the global dataset of DNA barcode sequences (i.e. including samples not generated in this study). We repeated all tree-building, genetic distance, and clustering analyses for a second, expanded dataset that combined the 96 adult reference sequences with 130 sequences from immature ticks collected from birds.

### Species-level associations between ticks and wild birds in Panama

We used the identifications of immature ticks from STRIBC bird specimens to further examine species-specific bird-tick associations in Panama. First, we assessed host-specificity by examining the correlation between the frequency of occurrence of a given tick species (number of birds infested by that species) and the diversity of avian host species (number of host species) for all tick species recovered in our dataset; if immature ticks are non-host specific, this correlation should be strong. While this approach provides one measurement of host–tick specificity, it potentially overlooks the role of ecological filtering by hosts for particular parasite species [41], which we tested for by examining differences in the frequency of parasitism by host ecological traits for each tick species identified using COI barcodes using *G*-tests of independence. We visualized the interaction between immature tick species and specific avian host species via quantitative interaction networks created using the bipartite package following the authors’ instructions [62] in the R statistical application [63]. For those birds that had multiple ticks identified by DNA barcoding we scored each tick species separately but only once in the data matrix.

Finally, to estimate the proportion and distribution of the total Panamanian tick species pool that might depend on birds as host vertebrates for immature life stages, we estimated the total species richness of ticks that parasitize wild birds in Panama through species accumulation curves generated in the EstimateS software package [64]. When sampling is exhaustive, the species accumulation curve should reach an asymptote. However, even non-exhaustive sampling can still yield sufficient data to provide a reasonable estimate of the true species richness, which can be assessed by observing an asymptote in the statistical estimate of species richness [65]. We generated species-accumulation curves (SACs) for the 130 ticks where we were successfully able to generate DNA barcodes using both the adult reference library cluster-indicated taxonomy, and using the BIN numerical taxonomy generated in the BOLD database. In both cases, we used the Chao1 species richness estimator [66], which attempts to non-parametrically correct the observed species richness as a function of the proportion of species observed exactly once or twice in the dataset. Mean Chao1 values were obtained from 100 reshuffles of our data set with replacement in EstimateS; sampling with replacement being critical in order to account for sampling error.

## Supporting Information

S1 Fig Phylogenetic tree of adult reference library. Neighbor-joining tree of 96 adult ticks based on COI DNA barcode codes.
(PDF)

S2 Fig Species accumulation curve (SAC) for immature ticks recovered from Panamanian wild birds based BIN numerical taxonomy. Black line = *S*, mean observed species richness; solid gray line = *Ŝ*, mean Chao1 *S* estimate; dotted gray lines = 95% upper and lower confidence limits (CI) for *Ŝ*. As Chao1 is downward biased, the 95% lower CI is probably not useful. Fairchild et al. [20] estimated that 37 species of hard ticks occur in Panama.
(PDF)

S1 Table Collecting data for bird vouchers sampled as potential hosts of ticks. (XLS)

S2 Table Geographical coordinates for bird sampling locations. (XLSX)

S3 Table Specimen data for adult ticks used to create adult reference library. (XLSX)

## References

[1] Rappole JH, Hubálek Z. Migratory birds and West Nile virus. J Appl Microbiol. 2003;94: 47–58. 10.1046/j.1365-2672.94.s1.6.x 12675936

[2] Kilpatrick AM, Chmura AA, Gibbons DW, Fleischer RC, Marra PP, Daszak P. Predicting the global spread of H5N1 avian influenza. Proc Natl Acad Sci. 2006;103: 19368–19373. 10.1073/pnas.0609227103 17158217 PMC1748232

[3] Ogden NH, Lindsay LR, Hanincová K, Barker IK, Bigras-Poulin M, Charron DF, et al Role of migratory birds in introduction and range expansion of *Ixodes scapularis* ticks and of *Borrelia burgdorferi* and *Anaplasma phagocytophilum* in Canada. Appl Environ Microbiol. 2008;74: 1780–1790. 10.1128/AEM.01982-07 18245258 PMC2268299

[4] Ginsberg HS, Buckley PA, Balmforth MG, Zhioua E, Mitra S, Buckley FG. Reservoir competence of native North American birds for the Lyme disease spirochete, *Borrelia burgdorferi*. J Med Entomol. 2005;42: 445–449. 10.1093/jmedent/42.3.445 15962798

[5] Hamer SA, Goldberg TL, Kitron UD, Brawn JD, Anderson TK, Loss SR, et al Wild birds and urban ecology of ticks and tick-borne pathogens, Chicago, Illinois, USA, 2005–2010. Emerg Infect Dis. 2012;18: 1589–1595. 10.3201/eid1810.120511 23017244 PMC3471635

[6] Lindeborg M, Barboutis C, Ehrenborg C, Fransson T, Jaenson TGT, Lindgren P-E, et al Migratory birds, ticks, and Crimean-Congo hemorrhagic fever virus. Emerg Infect Dis. 2012;18: 2095–2097. 10.3201/eid1812.120718 23171591 PMC3557898

[7] Jongejan F, Uilenberg G. The global importance of ticks. Parasitology. 2004;129: S3–S14. 10.1017/S0031182004005967 15938502

[8] Léger E, Vourc’h G, Vial L, Chevillon C, McCoy KD. Changing distributions of ticks: causes and consequences. Exp Appl Acarol. 2013;59: 219–244. 10.1007/s10493-012-9615-0 23015121

[9] Parola P, Socolovschi C, Jeanjean L, Bitam I, Fournier P-E, Sotto A, et al Warmer weather linked to tick attack and emergence of severe rickettsioses. PLoS Negl Trop Dis. 2008;2: e338 10.1371/journal.pntd.0000338 19015724 PMC2581602

[10] Randolph SE. To what extent has climate change contributed to the recent epidemiology of tick-borne diseases? Vet Parasitol. 2010;167: 92–94. 10.1016/j.vetpar.2009.09.011 19833440

[11] Parola P, Paddock CD, Socolovschi C, Labruna MB, Mediannikov O, Kernif T, Abdad MY, Stenos J, Bitam I, Fournier PE, Raoult D. Update on tick-borne rickettsioses around the world: a geographic approach. Clin Microbiol Rev. 2013; 26(4):657–702. 10.1128/CMR.00032-13 24092850 PMC3811236

[12] Dantas-Torres F. Rocky Mountain spotted fever. Lancet Infect Dis. 2007;7: 724–732. 10.1016/S1473-3099(07)70261-X 17961858

[13] Nava S, Beati L, Labruna MB, Cáceres AG, Mangold AJ, Guglielmone AA. Reassessment of the taxonomic status of *Amblyomma cajennense* (Fabricius, 1787) with the description of three new species, *Amblyomma tonelliae n*. *sp*., *Amblyomma interandinum n*. *sp*. and *Amblyomma patinoi n*. *sp*., and reinstatement of *Amblyomma mixtum* Koch, 1844, and *Amblyomma sculptum* Berlese, 1888 (Ixodida: Ixodidae). Ticks Tick-Borne Dis. 2014;5: 252–276. 10.1016/j.ttbdis.2013.11.004 24556273

[14] Pinter A, Labruna MB. Isolation of *Rickettsia rickettsii* and *Rickettsia bellii* in cell culture from the tick *Amblyomma aureolatum* in Brazil. Ann N Y Acad Sci. 2006;1078: 523–529. 10.1196/annals.1374.103 17114770

[15] Estripeaut D, Aramburú MG, Sáez-Llorens X, Thompson HA, Dasch GA, Paddock CD, et al Rocky Mountain spotted fever, Panama. Emerg Infect Dis. 2007;13: 1763–1765. 10.3201/eid1311.070931 18217566 PMC3375809

[16] Tribaldos M, Zaldivar Y, Bermudez S, Samudio F, Mendoza Y, Martinez AA, et al Rocky Mountain spotted fever in Panama: a cluster description. J Infect Dev Ctries. 2011;5: 737–741. 21997944 10.3855/jidc.2189

[17] Argüello AP, Hun L, Rivera P, Taylor L. A fatal urban case of Rocky Mountain spotted fever presenting an eschar in San Jose, Costa Rica. Am J Trop Med Hyg. 2012;87: 345–348. 10.4269/ajtmh.2012.12-0153 22855769 PMC3414575

[18] Hidalgo M, Miranda J, Heredia D, Zambrano P, Vesga JF, Lizarazo D, et al Outbreak of Rocky Mountain spotted fever in Córdoba, Colombia. Mem Inst Oswaldo Cruz. 2011;106: 117–118. 21340366 10.1590/s0074-02762011000100019

[19] Eremeeva ME, Dasch GA. Challenges posed by tick-borne rickettsiae: eco-epidemiology and public health implications. Front Public Health. 2015;3: 55 10.3389/fpubh.2015.00055 25954738 PMC4404743

[20] Fairchild GB, Kohla GM, Tipton VJ. The ticks of Panama (Acarina: Ixodoidea) Ectoparasites of Panama. Chicago: Field Museum of Natural History; 1966 pp. 167–219.

[21] Ogrzewalska M, Martins T, Capek M, Literak I, Labruna MB. A *Rickettsia parkeri*-like agent infecting *Amblyomma calcaratum* nymphs from wild birds in Mato Grosso do Sul, Brazil. Ticks Tick-Borne Dis. 2013;4: 145–147. 10.1016/j.ttbdis.2012.07.001 23238243

[22] Ogrzewalska M, Uezu A, Labruna MB. Ticks (Acari: Ixodidae) infesting wild birds in the eastern Amazon, northern Brazil, with notes on rickettsial infection in ticks. Parasitol Res. 2010;106: 809–816. 10.1007/s00436-010-1733-1 20140452

[23] Ogrzewalska M, Pacheco RC, Uezu A, Richtzenhain LJ, Ferreira F, Labruna MB. Ticks (Acari: Ixodidae) infesting birds in an Atlantic rain forest region of Brazil. J Med Entomol. 2009;46: 1225–1229. 19769058 10.1603/033.046.0534

[24] Ogrzewalska M, Uezu A, Jenkins CN, Labruna MB. Effect of forest fragmentation on tick infestations of birds and tick infection rates by rickettsia in the Atlantic forest of Brazil. EcoHealth. 2011;8: 320–331. 10.1007/s10393-011-0726-6 22173291

[25] Pacheco RC, Arzua M, Nieri-Bastos FA, Moraes-Filho J, Marcili A, Richtzenhain LJ, et al Rickettsial infection in ticks (Acari: Ixodidae) collected on birds in southern Brazil. J Med Entomol. 2012;49: 710–716. 22679880 10.1603/me11217

[26] Ogrzewalska M, Literak I, Cardenas-Callirgos JM, Capek M, Labruna MB. *Rickettsia bellii* in ticks *Amblyomma varium* Koch, 1844, from birds in Peru. Ticks Tick-Borne Dis. 2012;3: 254–256. 10.1016/j.ttbdis.2012.05.003 22809734

[27] Labruna MB, Sanfilippo LF, Demetrio C, Menezes AC, Pinter A, Guglielmone AA, et al Ticks collected on birds in the state of São Paulo, Brazil. Exp Appl Acarol. 2007;43: 147–160. 10.1007/s10493-007-9106-x 17882514

[28] Labruna MB, McBride JW, Bouyer DH, Camargo LMA, Camargo EP, Walker DH. Molecular evidence for a spotted fever group *Rickettsia* species in the tick *Amblyomma longirostre* in Brazil. J Med Entomol. 2004;41: 533–537. 15185961 10.1603/0022-2585-41.3.533

[29] Labruna MB, Mattar V S, Nava S, Bermudez S, Venzal JM, Dolz G, et al Rickettsioses in Latin America, Caribbean, Spain and Portugal. Rev MVZ Córdoba. 2011;16: 2435–2457.

[30] Martins TF, Onofrio VC, Barros-Battesti DM, Labruna MB. Nymphs of the genus *Amblyomma* (Acari: Ixodidae) of Brazil: descriptions, redescriptions, and identification key. Ticks Tick-Borne Dis. 2010;1: 75–99. 10.1016/j.ttbdis.2010.03.002 21771514

[31] Bermúdez C. SE, Castro A, Esser H, Liefting Y, García G, Miranda RJ. Ticks (Ixodida) on humans from central Panama, Panama (2010–2011). Exp Appl Acarol. 2012;58: 81–88. 10.1007/s10493-012-9564-7 22544074

[32] Marini MA, Reinert BL, Bornschein MR, Pinto JC. Ecological correlates of ectoparasitism on Atlantic Forest birds, Brazil. Ararajuba. 1996;4: 93–102.

[33] Rocha LA, Aleixo A, Allen G, Almeda F, Baldwin CC, Barclay MVL, et al Specimen collection: an essential tool. Science. 2014;344: 814–815. 10.1126/science.344.6186.814 24855245

[34] Ogrzewalska M, Literák I, Capek M, Sychra O, Calderón VÁ, Rodríguez BC, et al Bacteria of the genus *Rickettsia* in ticks (Acari: Ixodidae) collected from birds in Costa Rica. Ticks Tick-Borne Dis. 2015;6: 478–482. 10.1016/j.ttbdis.2015.03.016 25869035

[35] Guglielmone AA, Estrada-Peña A, Mangold AJ, Barros-Battesti DM, Labruna MB, Martins JR, et al *Amblyomma aureolatum* (Pallas, 1772) and *Amblyomma ovale* Koch, 1844 (Acari: Ixodidae): hosts, distribution and 16S rDNA sequences. Vet Parasitol. 2003;113: 273–288. 12719142 10.1016/s0304-4017(03)00083-9

[36] Nava S, Szabó MPJ, Mangold AJ, Guglielmone AA. Distribution, hosts, 16S rDNA sequences and phylogenetic position of the Neotropical tick *Amblyomma parvum* (Acari: Ixodidae). Ann Trop Med Parasitol. 2008;102: 409–425. 10.1179/136485908X278883 18577332

[37] McCoy KD, Léger E, Dietrich M. Host specialization in ticks and transmission of tick-borne diseases: a review. Front Cell Infect Microbiol. 2013;3 10.3389/fcimb.2013.00057 PMC3790072 24109592

[38] Wells K, O’Hara RB, Pfeiffer M, Lakim MB, Petney TN, Durden LA. Inferring host specificity and network formation through agent-based models: tick–mammal interactions in Borneo. Oecologia. 2012;172: 307–316. 10.1007/s00442-012-2511-9 23108423

[39] Madinah A, Abang F, Mariana A, Abdullah M t., Mohd-Azlan J. Interaction of ectoparasites-small mammals in tropical rainforest of Malaysia. Community Ecol. 2014;15: 113–120. 10.1556/ComEc.15.2014.1.12

[40] Nava S, Guglielmone A. A meta-analysis of host specificity in Neotropical hard ticks (Acari: Ixodidae). Bull Entomol Res. 2013;103: 216–224. 10.1017/S0007485312000557 22954015

[41] Combes C. Parasitism: the ecology and evolution of intimate interactions. Chicago: University of Chicago Press; 2001.

[42] Poulin R. Evolutionary ecology of parasites, 2nd ed. Princeton: Princeton University Press; 2011.

[43] Hebert PDN, Penton EH, Burns JM, Janzen DH, Hallwachs W. Ten species in one: DNA barcoding reveals cryptic species in the neotropical skipper butterfly Astraptes fulgerator. Proc Natl Acad Sci U S A. 2004;101: 14812–14817. 10.1073/pnas.0406166101 15465915 PMC522015

[44] Bermúdez SE, Miranda RJ, Smith D. Ticks species (Ixodida) in the Summit Municipal Park and adjacent areas, Panama City, Panama. Exp Appl Acarol. 2010;52: 439–448. 10.1007/s10493-010-9374-8 20585838

[45] de Rodaniche E. C., Rodaniche A. Spotted fever in Panama; isolation of the etiologic agent from a fatal case. Am J Trop Med Hyg. 1950;30: 511–517. 15425741 10.4269/ajtmh.1950.s1-30.511

[46] Bermúdez CSE, Zaldívar AY, Spolidorio MG, Moraes-Filho J, Miranda RJ, Caballero CM, et al Rickettsial infection in domestic mammals and their ectoparasites in El Valle de Antón, Coclé, Panamá. Vet Parasitol. 2011;177: 134–138. 10.1016/j.vetpar.2010.11.020 21144663

[47] De Rodaniche EC. Natural infection of the tick, *Amblyomma cajennense*, with *Rickettsia rickettsii* in Panama. Am J Trop Med Hyg. 1953;2: 696–699. 13065638 10.4269/ajtmh.1953.2.696

[48] Estrada-Peña A, Guglielmone AA, Mangold AJ. The distribution and ecological “preferences” of the tick *Amblyomma cajennense* (Acari: Ixodidae), an ectoparasite of humans and other mammals in the Americas. Ann Trop Med Parasitol. 2004;98: 283–292. 10.1179/000349804225003316 15119974

[49] Bermúdez SE, Eremeeva ME, Karpathy SE, Samudio F, Zambrano ML, Zaldivar Y, et al Detection and identification of rickettsial agents in ticks from domestic mammals in eastern Panama. J Med Entomol. 2009;46: 856–861. 19645289 10.1603/033.046.0417

[50] Eremeeva ME, Karpathy SE, Levin ML, Caballero CM, Bermudez S, Dasch GA, et al Spotted fever rickettsiae, *Ehrlichia* and *Anaplasma*, in ticks from peridomestic environments in Panama. Clin Microbiol Infect Off Publ Eur Soc Clin Microbiol Infect Dis. 2009;15 Suppl 2: 12–14. 10.1111/j.1469-0691.2008.02638.x 19456809

[51] Cohen EB, Auckland LD, Marra PP, Hamer SA. Avian migrants facilitate invasions of Neotropical ticks and tick-borne pathogens into the United States. Appl Environ Microbiol. 2015;81: 8366–8378. 10.1128/AEM.02656-15 26431964 PMC4644638

[52] Apperson CS, Engber B, Nicholson WL, Mead DG, Engel J, Yabsley MJ, et al Tick-borne diseases in North Carolina: is “*Rickettsia amblyommii*” a possible cause of rickettsiosis reported as Rocky Mountain spotted fever? Vector Borne Zoonotic Dis Larchmt N. 2008;8: 597–606. 10.1089/vbz.2007.0271 18447622

[53] Miranda J, Portillo A, Oteo JA, Mattar S. *Rickettsia sp*. strain colombianensi (Rickettsiales: Rickettsiaceae): a new proposed *Rickettsia* detected in *Amblyomma dissimile* (Acari: Ixodidae) from iguanas and free-living larvae ticks from vegetation. J Med Entomol. 2012;49: 960–965. 22897060 10.1603/me11195

[54] Romer Y, Seijo AC, Crudo F, Nicholson WL, Varela-Stokes A, Lash RR, et al *Rickettsia parkeri* Rickettsiosis, Argentina. Emerg Infect Dis. 2011;17: 1169–1173. 10.3201/eid1707.101857 21762568 PMC3381406

[55] Robinson WD, Brawn JD, Robinson SK. Forest bird community structure in central Panama: influence of spatial scale and biogeography. Ecol Monogr. 2000;70: 209–235. 10.1890/0012-9615(2000)070[0209:FBCSIC]2.0.CO;2

[56] Clayton DH, Drown DM. Critical evaluation of five methods for quantifying chewing lice (Insecta: Phthiraptera). J Parasitol. 2001;87: 1291–1300. 10.1645/0022-3395(2001)087[1291:CEOFMF]2.0.CO;2 11780812

[57] Onofrio V, Labruna M, Pinter A, Giacomin F, Barros-Battesti D. Comentários e chaves as espécies do gênero *Amblyomma* In: Barros-Battesti D, Arzua M, Bechara G, editors. Carrapatos de Importância Médico-Veterinária da Região Neotropical: Un guia ilustrado para identificação de espécie. São Paulo: Instituto Butantan; pp. 53–113.

[58] Ratnasingham S, Hebert PDN. BOLD: The Barcode of Life Data System (http://www.barcodinglife.org). Mol Ecol Notes. 2007;7: 355–364. 10.1111/j.1471-8286.2007.01678.x 18784790 PMC1890991

[59] Kumar NP, Rajavel AR, Natarajan R, Jambulingam P. DNA barcodes can distinguish species of Indian mosquitoes (Diptera: Culicidae). J Med Entomol. 2007;44: 1–7. 17294914 10.1603/0022-2585(2007)44[1:dbcdso]2.0.co;2

[60] Tamura K, Peterson D, Peterson N, Stecher G, Nei M, Kumar S. MEGA5: molecular evolutionary genetics analysis using maximum likelihood, evolutionary distance, and maximum parsimony methods. Mol Biol Evol. 2011;28: 2731–2739. 10.1093/molbev/msr121 21546353 PMC3203626

[61] Ratnasingham S, Hebert PDN. A DNA-based registry for all animal species: the barcode index number (BIN) system. PloS One. 2013;8: e66213 10.1371/journal.pone.0066213 23861743 PMC3704603

[62] Dormann CF, Fründ J, Blüthgen N, Gruber B. Indices, Graphs and Null Models: Analyzing Bipartite Ecological Networks [Internet]. 2009. Available: http://goedoc.uni-goettingen.de/goescholar/handle/1/5837

[63] R Development Core Team. R Development Core Team (2013). R: A language and environment for statistical computing R Foundation for Statistical Computing, Vienna, Austria ISBN 3-900051-07-0, URL http://www.R-project.org. R Foundation for Statistical Computing, Vienna, Austria; 2013.

[64] Colwell R. EstimateS: Statistical estimation of species richness and shared species from samples. Version 9. User’s Guide and application published at: http://purl.oclc.org/estimates. 2013.

[65] Colwell RK, Coddington JA. Estimating terrestrial biodiversity through extrapolation. Philos Trans R Soc Lond B Biol Sci. 1994;345: 101–118. 10.1098/rstb.1994.0091 7972351

[66] Chao A. Estimating the population size for capture-recapture data with unequal catchability. Biometrics. 1987;43: 783–791. 3427163

