# Supplementary figures and images for: Molecular Ecological Insights into Neotropical Bird–Tick Interactions
Source: PLoS One. 2016 May 20;11(5):e0155989. doi: 10.1371/journal.pone.0155989 (PMC4874597; doi:10.1371/journal.pone.0155989)

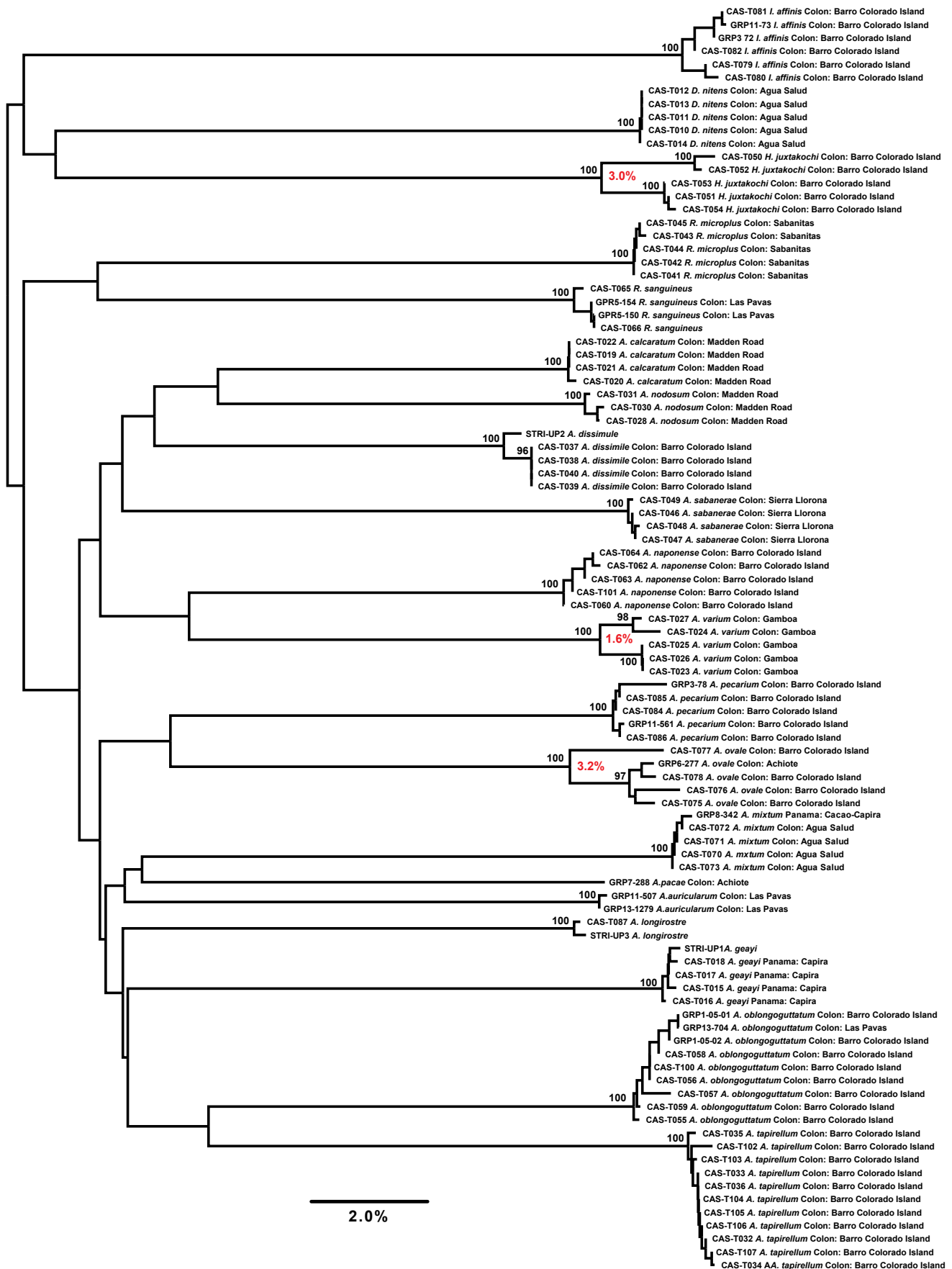

Supplement: S1 Fig — Neighbor-joining tree of 96 adult ticks based on COI DNA barcode codes. (PDF) [file pone.0155989.s001.pdf]

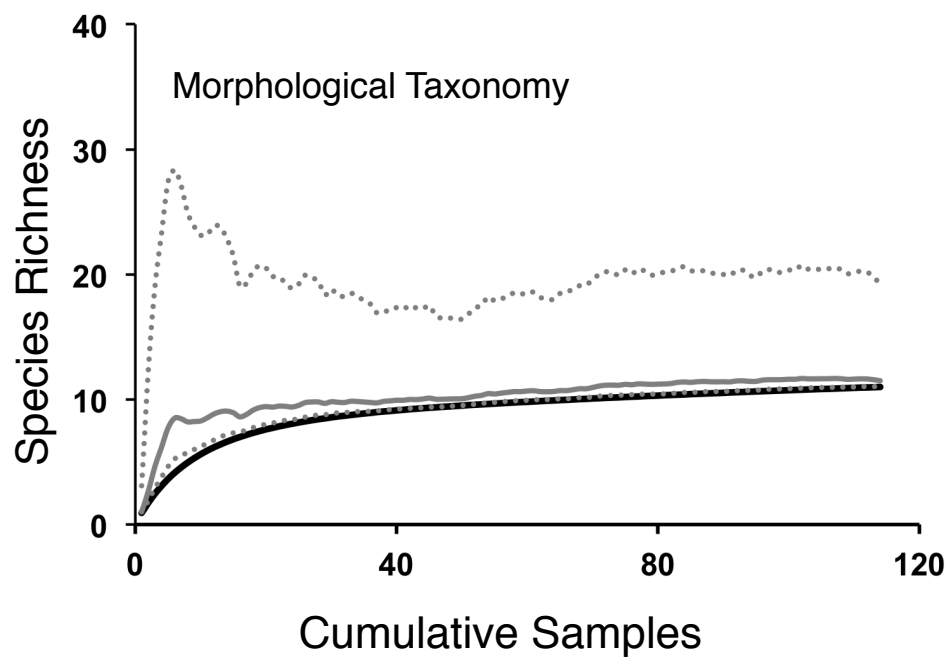

Supplement: S2 Fig — Black line = S, mean observed species richness; solid gray line = Ŝ, mean Chao1 S estimate; dotted gray lines = 95% upper and lower confidence limits (CI) for Ŝ. As Chao1 is downward biased, the 95% lower CI is probably not useful. Fairchild et al. [20] estimated that 37 species of hard ticks occur in Panama. (PDF) [file pone.0155989.s002.pdf]
